# Retinoic acid exacerbates chlorpyrifos action in ensuing adipogenic differentiation of C3H10T½ cells in a GSK3β dependent pathway

**DOI:** 10.1371/journal.pone.0173031

**Published:** 2017-03-14

**Authors:** Harkirat Singh Sandhu, A. J. S. Bhanwer, Sanjeev Puri

**Affiliations:** 1 Department of Human Genetics, Guru Nanak Dev University, Amritsar, Punjab, India; 2 Centre for Stem Cell Tissue Engineering and Biomedical Excellence, Panjab University, Chandigarh, India; 3 Biotechnology Branch, University Institute of Engineering and Technology, Panjab University, Chandigarh, India; University of Texas at Austin Dell Medical School, UNITED STATES

## Abstract

The cell differentiation can be exploited as a paradigm to evaluate the effects of noxious chemicals, on human health, either alone or in combinations. In this regard, the effect of a known cell differentiation agent, retinoic acid (RA) was analyzed in the presence of a noxious chemical chlorpyrifos (CPF), an organophosphate (OP), the receptors of which have recently been localized to mesenchymal stem cells (MSCs). The observed imbalance of adipogenic to skeletal differentiation by CPF together with conundrum about adipogenic potential of RA prompted us to delineate their combinatorial effects on C_3_H_10_T½MSC-like undifferentiated cells. Based on MTT assay, the cellular viability was retained by CPF at concentrations ranging from 0.01–50μM, beyond which it caused cytotoxicity. These non-toxic concentrations also mildly interfered with adipogenesis of C_3_H_10_T½ cells following exposure to adipogenic cocktail. However, upon exposure to RA alone, these MSCs adopted elongated morphology and accumulated lipid vesicles, by day 20, as discerned by phase-contrast and transmission electron microscopy (TEM), in concert with enhanced Oil Red O stained cells. This effect got strongly augmented upon exposure to combination of CPF and RA in a dose-dependent manner. Simultaneous up-regulation in *perilipin-1 (PLIN1)* and *adipsin (ADN)* genes, additionally reiterated the adipogenic differentiation. Mechanistically, GSK3β pathway was found to be a major player, whereby inhibiting it with lithium chloride (LiCl) resulted in complete blockage of lipid accumulation, accompanied by complete down regulation of *PLIN1* and *ADN* gene expression. In conclusion, these observations for the first time, lend evidence that exposure of CPF accompanied by RA directs commitment of C_3_H_10_T½ cells to adipogenic differentiation through a process involving a crosstalk at GSK3β signaling.

## Introduction

Constant human exposure to noxious xenobiotics like pesticides is indispensable due to their widespread agricultural and domestic usage often leading to adverse health effects [1, 2]. Broadly, being divided into three groups’ viz. organophosphates (OP), organochlorines (OC) and carbamates (CB), the former is the most frequently used, accounting for more than 50% of poisoning cases [3, 4]. Besides being prevalently used in agriculture, these pesticides also find their use in household as pet shampoos, and in control of insects in houses and as lawn sprays [5, 6]. This suggests an unprecedented increase in the exposure of OP pesticides with parallel increase in the potential toxic effects on non-target organisms. Thus, in order to assess the potential risk involved in exposure to pesticides alone or as complex mixtures, an *in vitro* cell-based test can help provide useful information regarding danger to human health. Since, cell differentiation is a biological process of fundamental importance in developing and adult organisms. In this paper, we propose a cell-based test system for continuous, label-free monitoring of the effect of test substances on stem cell differentiation.

Among many different OPs, chlorpyrifos (CPF) is the one most prevalently used [7–9]. The mechanism of action of CPF primarily involves blocking the activity of acetyl cholinesterase (AChE), thereby exerting neurotoxic effects [10]. Besides its neurotoxic effects, the effects on other cell types are attributed to presence of AChE receptors on different non-target cell types viz. blood cells [11], osteoblasts [12, 13] and mesenchymal stem cells (MSCs) [14, 15]. The situation becomes excessively alarming owing to the combined exposure, knowingly or unknowingly to other chemicals which predispose one over the other to different fate and hence, the unwanted effects as well. A suspected link between OP pesticides and reduced bone formation in humans has been reported [16]. The expression of high levels of AChE in bone-forming osteoblasts and their progenitors would support an effect of AChE inhibitors on these cells [17]. The MSCs being multipotent cells are capable of self-renewal and rapid expansion and possess an inherent potential of lineage commitment towards adipocytes, osteocytes, myocytes and chondrocytes [18]. The localization of OP responsive receptors on MSCs thus raises concerns about cell differentiation fate. Compromised bone formation upon chronic exposure to OPs, seen both at the cellular and tissue level [16] thus, suggest their effects on bone progenitors. Besides, these have also been shown to induce hypothyroidism and euthyroidism, where both these conditions are related with abnormal weight gain, thus anticipating their effect on the adipose tissue which is an active endocrine organ involved in energy homeostasis [19] and also rich in MSCs [20]. Both osteoblasts (bone cells) and adipocytes (fat cells) originating from MSCs actually represent mechanistically coupled arms of bone remodeling and have also been linked to osteoporosis. Their reciprocal relationship resulting in increased bone marrow adiposity increases the susceptibility to osteoporotic fractures by reducing bone mass [21–25]. Thus, the variations in MSC differentiation upon pesticide exposure might be involved in causing bone metabolic diseases. However, incongruity as for the adipogenesis being a differentiation lineage of MSCs following OP pesticides has remained a puzzle. To sort out this conundrum, we exploited a known molecule of cellular differentiation, the retinoic acid (RA). With regard to adipogenesis, RA is involved in enhancing lipid accumulation in AML-1 preadipocytes [26], preadipocytes from bovine white adipose tissue [27], Ob1771 mouse fibroblasts [28], bone-marrow derived MSCs [29] and murine embryonic stem cells [30]. Since the time of its discovery, it has been considered as a model compound for inducing osteoporosis by causing a reduction in bone mass through increased bone resorption via osteoclast differentiation [31, 32]. In addition, it is a powerful teratogen and has been known to play a vital role in neuronal differentiation [33, 34], partly pointing towards OP-like effects.

The individualistic effects of CPF and RA have been shown on human and mouse neuroprogenitor cell lines, with a focus being on proliferative and apoptotic changes, where both caused a 50% decrease in cellular proliferation [35]. Another recent study has demonstrated the effect of OP pesticides on RA mediated neuronal differentiation of NT2 cells [36], however, none of the studies till date have ever reported their combinatorial effect on MSC differentiation. Hence, in the present study, the undifferentiated and multipotent C_3_H_10_T½ cells were exploited to investigate the interaction of CPF and RA with respect to their potential of adipogenic differentiation in MSCs, together with elucidation of the underlying mechanism mediating this effect. These cells serve as a model system for bone marrow-derived MSCs by providing a homogenous and phenotypically stable cell population.

## Materials and methods

### Reagents

C_3_H_10_T½ undifferentiated murine cells with MSC-like properties were procured from National Centre for Cell Science (NCCS), Pune, India. All the chemicals used were of molecular and analytical grade. RA, dexamethasone, 3-isobutyl-1-methylxanthine (IBMX), insulin, Oil Red O and lithium chloride (LiCl) were purchased from Sigma-Aldrich, Mumbai, India. The cell culture reagents employed were purchased from Himedia, Mumbai, India. Technical grade CPF (98% purity) was procured from Gharda Chemicals Ltd., Mumbai, India. Primers used for gene expression studies were bought from, Sigma-Aldrich, Bangalore, India.

### Cell culture and differentiation

C3H10T½ cells were cultured in Dulbecco’s Modified Eagle’s Medium (DMEM) containing 4.5% glucose and L-glutamine supplemented with 10% (v/v) heat-inactivated fetal bovine serum (FBS) and 100 U/ml penicillin-streptomycin at 37°C in a humidified atmosphere containing 5% CO_2_. The cells were passaged using 0.25% trypsin containing 0.02% ethylene diamine tetra acetic acid (EDTA) after reaching a confluence of ~80–90%. They were used between passages 2–7 for the experiments described below. Cells were seeded at a concentration of 1.5 x 10^5^ cells/well of the 6-well plate in DMEM containing 5% FBS. After reaching a confluence of 50–60%, cells were subjected to 2 μM RA treatment for 20 days. Concentrated stock solution of 1 M RA was prepared in dimethyl sulfoxide (DMSO), sterile filtered through a 0.22 μm syringe filter and stored in dark at -20°C. Working solutions of 2 μM were further prepared by serial dilutions. Similarly, serially diluted CPF across a range of 0.01–50 μM employed in the study was added along with RA at day 0 and continued until the experimental endpoint. Media changes were made every 3 days with fresh addition of all the doses. For inhibitory studies, 1 hr treatment with LiCl was given prior to addition of the differentiation doses at day 0, and later refreshed along with the doses. The vehicle control contained DMSO alone, with its final concentration adjusted to 0.005%. Each treatment was performed in triplicate. For the conventional adipogenic differentiation, post-confluent cells were induced with the hormonal cocktail comprising of 1 μM dexamethasone, 0.5 mM IBMX and 10 ng/ml insulin in full growth media (FGM) supplemented with 10% FBS and re-fed with fresh media every 3 days. Phase-contrast images were taken daily at 100x magnification in Radical RTC-7 phase contrast inverted microscope using the Pro Jenoptix software.

### Oil Red O staining

For staining the cytoplasmic lipid vesicles, Protocol of Janderova *et al* and Ramirez-Zacarias *et al*was followed [37, 38]. After aspirating the media, cells were washed with phosphate buffered saline (PBS) and fixed in 10% formalin at 37°C for 1 hr. Cells were then washed with 60% isopropanol and allowed to dry completely. 1.5 ml Oil Red O staining solution was added to the wells for a period of 30 min. For preparing the staining solution, 0.35 g Oil Red O was dissolved in 100 ml isopropanol, stirred overnight on a magnetic stirrer, filtered with 0.22 μm syringe filter and stored at 4°C. Working solution for immediate use was made by diluting the stock solution with distilled water in a 6:4 ratio, mixing and incubating at room temperature for 20 min. After incubation with the Oil Red O working solution, excess stain was washed 3–4 times with PBS and counter-stained with hematoxylin for 1 min. The lipid vesicles were analyzed under bright field microscope at 100x magnification.

### Cell viability assay

The viability of the cells was evaluated using the MTT [3-(4,5-Dimethylthiazol-2-yl)-2,5-Diphenyltetrazolium Bromide] assay. Cells were seeded in 96-well microtiter plates at a concentration of 1x10^4^ cells/well in 100 μl of FGM containing 0.1% FBS. After reaching 50–60% confluence, the various doses were added and incubated for 24 hrs. 10 μl of 5 mg/ml MTT in PBS was added to each well and incubated for 3–4 hrs at 37°C. After the formation of purple colored formazan crystals, 100 μl of acidified isopropanol (0.1 N HCL in isopropanol) was added to dissolve the crystals by pipetting up and down until the content got homogenized. This was left in the dark for 10 min and absorbance read at 570 nm in an ELISA reader.

### RT PCR analysis

Total RNA was extracted from C3H10T½ cells on day 20 using the TRIzol reagent [39]. RNA abundance and integrity were assessed spectrophotometrically using the Nano Drop and absorbance read at 260 nm. 1 μg of this RNA was reverse transcribed using the Superscript III one-step RT PCR system, according to manufacturer’s instructions. Primers were designed using the Primer3 software as represented in Table 1. Except for housekeeping *β-actin* gene (35 cycles), the amplification of *perilipin-1 (PLIN1)* and *adipsin (ADN)* genes was performed for 33 cycles keeping the PCR condition uniform i.e. 94°C, 60°C and 68°C. The RT PCR products were run on 2% agarose gels stained with ethidium bromide and captured on the gel doc. Densitometric analysis was done using the ImageJ software. mRNA values were normalized to the expression level of the reference *β-*actin gene.

**Table 1:** Primers used for Reverse Transcriptase Polymerase Chain Reaction analysis.

Gene	Orientation	Primer sequence	Product Size (bp)	Accession number
***PLIN1***	Forward	5’-AGTCAGCGACAGCTTCTTCC-3’	247	NM_175640
	Reverse	5’-GGTGCCCCTTAAAAATTGGT-3’		
***ADN***	Forward	5’-TGCACAGCTCCGTGTACTTC-3’	294	NM_013459
	Reverse	5’-GCACATCATACCATCGCTTG-3’		
***β-actin***	Forward	5’-AAATCGTGCGTGACATCAAA-3’	447	NM_007393
	Reverse	5’-ACATCTGCTGGAAGGTGGAC-3’		

### Transmission electron microscopy studies

Differentiated cells upon trypsinization were fixed with a mixture of 2% paraformaldehyde and 2.5% glutaraldehyde for 2 hrs and processed for sample preparation. Post-fixation was performed in 1% (v/v) osmium tetroxide (OsO_4_). After undergoing a series of dehydration steps with graded alcohol concentrations, rinsing was done with propylene oxide and impregnation with epoxy resins. Ultrathin sections were made and stained with 4.8% uranyl acetate and lead citrate. Electron micrographs were taken using the JEM 2100 Jeol transmission electron microscope (TEM) and viewed with the iTEM software.

### Statistical analysis

Statistical analysis was performed using GraphPad Prism software. Data was presented as mean ± SEM (n = 3 or n = 6) and analyzed the stepwise multiple comparisons among the samples by one-way analysis of variance (ANOVA) with post-hoc test using Newman-Keuls method. Results were considered statistically significant at p<0.001 or 0.05 (α = 0.001 or 0.05, respectively) and denoted by an asterix (*).

## Results

### 1. Dose response of CPF and C_3_H_10_T½ MSC viability

**Fig 1:**
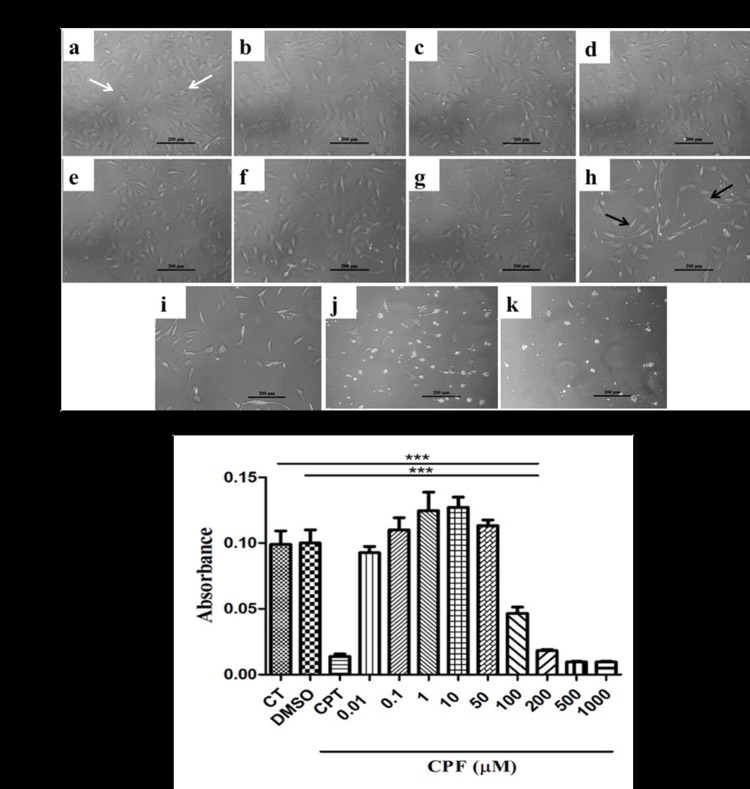
Concentration dependency of CPF on C_3_H_10_T½ MSC viability. **(A)** CPF at concentrations 0.01, 0.1, 1, 10, 50, 100, 200, 500, 1000 μM (c-k) retained the cell morphology similar to (a) control (CT, white arrowheads) and (b) vehicle (DMSO). The concentrations above 50 μM i.e. 100–1000 μM (h-k) affected the cell morphology (black arrow heads). Scale- 200 μm. **(B)** MTT assay for cell toxicity demonstrated cell viability retained till 50 μM, while a significant reduction in the cell viability was observed at concentrations beyond 50 μM (100–1000 μM). Hence, in majority of the subsequent experiments 50 μM concentration of CPF was used. Data was expressed as mean ± SEM (n = 6) and One-way ANOVA with Newman-Keuls Multiple Comparison Test performed for statistical analysis (*p<0.001).

For evaluating the toxicity of CPF, a dose responsive effect ranging from 0.01–1000 μM was tested on undifferentiated C_3_H_10_T½ cells for a period of 24 hrs. The photomicrograph (Fig 1A) demonstrates the cellular morphology following phase-contrast microscopy. Morphologically, the cells showed fibroblast-like appearance (white arrow heads) and remained adhered as flattened structures in control well (CT), also following vehicle (DMSO) treatment and CPF treatment at concentrations 0.01, 0.1, 1, 10 and 50 μM. However, further reaching higher concentrations of CPF to 100 μM or higher, 200, 500 and 1000 μM, gross changes in the cellular morphology were observed. At higher concentrations, significant decrease in cellular viability as seen by loss of fibroblast-like structures (black arrow heads) was observed. The concentrations 500 and 1000 μM were extremely toxic to the cells and caused immediate cell death.

Furthermore, to discern the extent of toxicity, cellular viability was analyzed employing MTT assay under same conditions as above. As a positive control, 1000 μM cisplatin (CPT) was used for comparing CPF cytotoxicity. As could be seen in Fig 1B, the concentrations ranging from 0.01–50 μM CPF remained refractory to cellular toxicity, as the cells retained their viability to same extent as could be seen for CT or following vehicle (DMSO) treatment. However, concentrations starting from 100 μM produced more than 50% loss in the cellular viability, which went further down at higher concentrations of CPF (200, 500, and 1000 μM). This effect was found to be akin to as observed in the cells treated with a known toxicant CPT. Hence, the concentrations ranging from 0.01–50 μM were preferred in majority of the subsequent experiments.

### 2. CPF interferes with adipogenic differentiation

**Fig 2:**
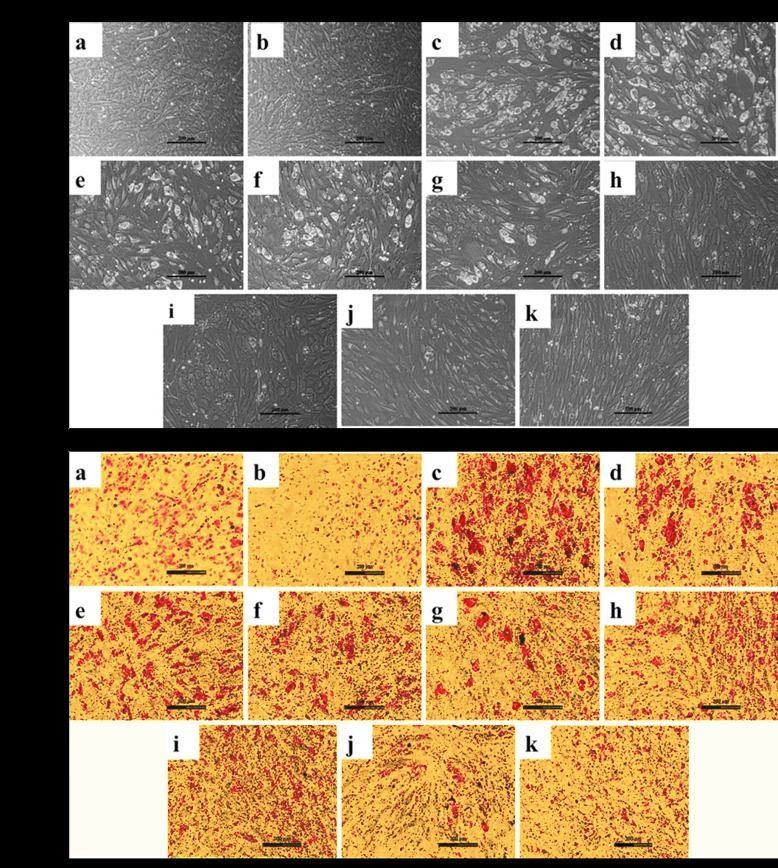
CPF alone prevented ability of adipogenic cocktail (DMI) towards differentiation of C_3_H_10_T½ cells to adipocytic lineage. **(A)** Phase-contrast micrographs indicated appearance of lipid vesicles (white) in (c) DMI and (d) DMI+ DMSO compared to (a) CT and (b) DMSO. Increasing concentrations of CPF (0.01, 0.1, 1, 10, 25, 50 and 100 μM) caused a gradual decrease in lipid vesicle accumulation of DMI-treated cells (e-k). **(B)** Oil Red O stained cells following adipogenic cocktail (DMI) treatment to C_3_H_10_T½ cells w.r.t CT and DMSO. CPF in a dose-dependent manner decreased the adipogenic differentiation (a-k, similar to above phase-contrast images). Scale- 200 μm.

Adipogenic differentiation of C_3_H_10_T½ cells, in the presence and absence of CPF, characterized by accumulation of intracellular cytoplasmic lipid vesicles (Fig 2A) and Oil Red O staining (Fig 2B) was analyzed continuously for a period of 21 days. As could be seen, the differentiated structures following exposure to adipogenic cocktail (DMI) comprising of dexamethasone, IBMX and insulin showed appearance of lipid vesicles in the cells with rounded morphology (Fig 2A) as well as red stained cells following Oil Red O staining (Fig 2B) compared to, fibroblast-like morphology as observed in the CT cells or the DMSO-treated cells. Co-addition of DMSO with DMI did not produce any effect as the extent of lipid vesicle accumulation and round cellular morphology together with the red colored staining of the cells remained same as those seen for DMI-treated cells.

However, the Oil Red O staining of CT and DMSO-treated cells showed no red stained cells. Increasing concentrations of CPF from 0.01–100 μM inhibited DMI-induced adipogenic differentiation in a dose-dependent manner as observed by decrease in lipid vesicle accumulation and changes in the cellular morphology as observed by phase-contrast microscope and further confirmed by reduction in the appearance of red stained cells following Oil Red O staining. These observations demonstrated that except for the lowest most concentration of CPF (0.01 μM), the concentrations varying from 0.1 μM, and above started to show anti-adipogenic effects with clear cut complete inhibition of adipogenic differentiation at 25 μM or higher.

### 3. CPF together with RA induces adipogenic differentiation

**Fig 3:**
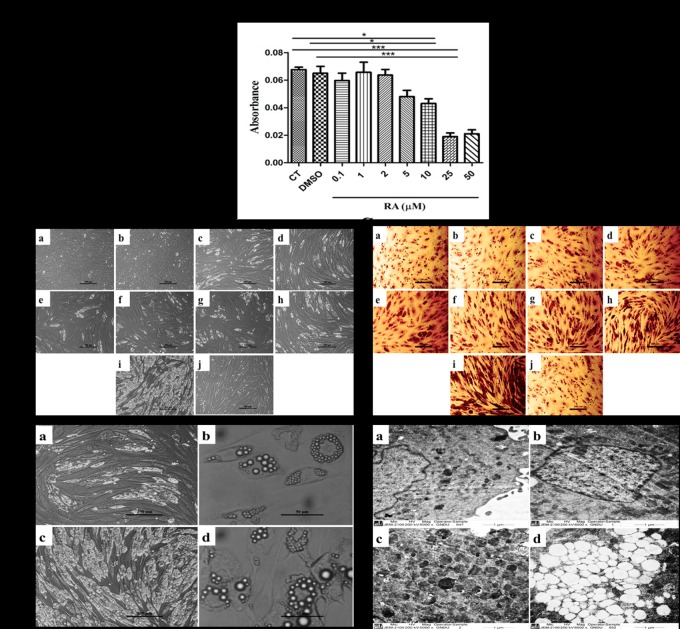
CPF augmented RA-induced differentiation of C_3_H_10_T½ cells to adipocytic lineage. **(A)** MTT assay shows cell viability retained by RA at concentrations ranging from 0.1–2 μM. Higher concentrations were found to be toxic. Hence, RA at concentration of 2 μM was used in all the subsequent experiments. Plotted values represent mean ± SEM (n = 3) and One-way ANOVA with Newman-Keuls Multiple Comparison Test performed for statistical analysis (*p<0.001). **(B)** Phase-contrast micrographs indicated appearance of lipid vesicles (white) in (c) RA in comparison to (a) CT, (b) DMSO and (j) CPF. Increasing concentrations of CPF (0.01, 0.1, 1, 10, 25, 50 μM) along with RA (2 μM) resulted in enhancement of lipid vesicles (d-i). Scale- 200 μm. **(C)** Oil Red O stained cells following above treatment. Scale- 50μm. These observations were further strengthened by **(D)** higher magnifications (a) RA (100x) (b) RA (400x) (c) RA+ CPF (100x) (d) RA+ CPF (400x). **(E)** 6000x transmission electron micrographs showing augmentation of lipid vesicles in combined treatment of CPF and RA (d) but considerably less in presence of RA alone (c). (a) CT and (b) CPF did not show appearance of any such lipid vesicles. Scale- 6000x.

Based on the above data, we sought to observe combined effects of CPF and RA. RA is a known differentiation agent by itself; therefore, no adipogenic differentiation medium was used in such studies. However, before observing the combined treatment effect, the MTT assay for assessing cell viability employing different concentrations of RA was carried out. As shown in Fig 3A, compared to CT and DMSO-treated cells, the RA levels were well tolerated by cells with concentrations varying from 0.1 μM upto 2 μM. Beyond this concentration, the cell viability reduced drastically where 25 and 50 μM RA proved significantly toxic to the cells. Hence, RA concentration corresponding to 2 μM was used in combination with varied concentrations of CPF i.e. 0.01–50 μM.

Fig 3B and 3C show the combined effects of RA (2 μM) and different concentrations of CPF (0.01–50 μM) on C_3_H_10_T½ cell morphology and Oil Red O staining, respectively. Compared to CT and DMSO-treated cells, the cells treated with RA showed a more elongated morphology. Moreover, the RA-treated cells were also positive for Oil Red O staining in comparison to CT or DMSO-treated cells. However, in presence of varying concentrations of CPF, 0.01–50 μM, with RA a gradual increase in the intracellular lipid accumulation (as seen by white vesicle-like structures) was seen by day 20 under the phase-contrast microscope.

In order to reassure the appearance of these vesicular-like structures, images at 100x and 400x magnification were taken to observe the effect of RA (2 μM) either alone or in combination with highest concentration of CPF (50 μM). The results in Fig 3D (100x) showed presence of whitish structures in RA-treated cells, which upon further magnification (400x) looked like vesicles accumulating in the cytoplasm of the cells. However, in the combined treatment group, there was further increase in these whitish appearing cells (100x), apparently the cells accumulated more lipid-like vesicles (400x).

The lipidic character of these cellular vesicles was reassured upon Oil Red O staining of these cells (Fig 3C). As shown in Fig 3C, the combined treatment of CPF and RA further augmented lipid accumulation in comparison to RA alone. The extent of Oil Red O staining in RA-treated cells increased further with increasing concentrations of CPF where, 50 μM CPF demonstrated unusually vast increase in lipid synthesis with ~80–90% cells being filled with lipid vesicles in comparison to RA treatment alone. However, 50 μM CPF alone did not accumulate cytoplasmic lipids, although morphological elongation was visible.

The fact that combined treatment increased the lipid accumulation; we further reiterated these observations by analyzing the overall cellular architecture through transmission electron microscopy (Fig 3E). Compared to CT, CPF (50 μM) or RA (2 μM) exposure, the combined effect of CPF and RA (50 μM + 2 μM) caused a huge increase in the accumulation of lipid vesicles.

### 4. Molecular mechanisms of adipogenic differentiation

#### 4.1 CPF together with RA induces adipogenesis through GSK3β signaling

The CPF (0.1–50 μM, Fig 2B) independently prevented differentiation of C_3_H_10_T½ cells to adipogenic lineage following exposure to adipogenic cocktail (DIM). However, it produced contrasting effects when co-treated with RA leading to augmentation of lipidogenesis over the RA treatment (Fig 3C). This prompted us to determine the underlying cause for such a variation. For this, the effects of a known regulator of adipogenesis viz. glycogen synthase kinase 3β (GSK3β) was studied employing its specific inhibitor LiCl.

**Fig 4:**
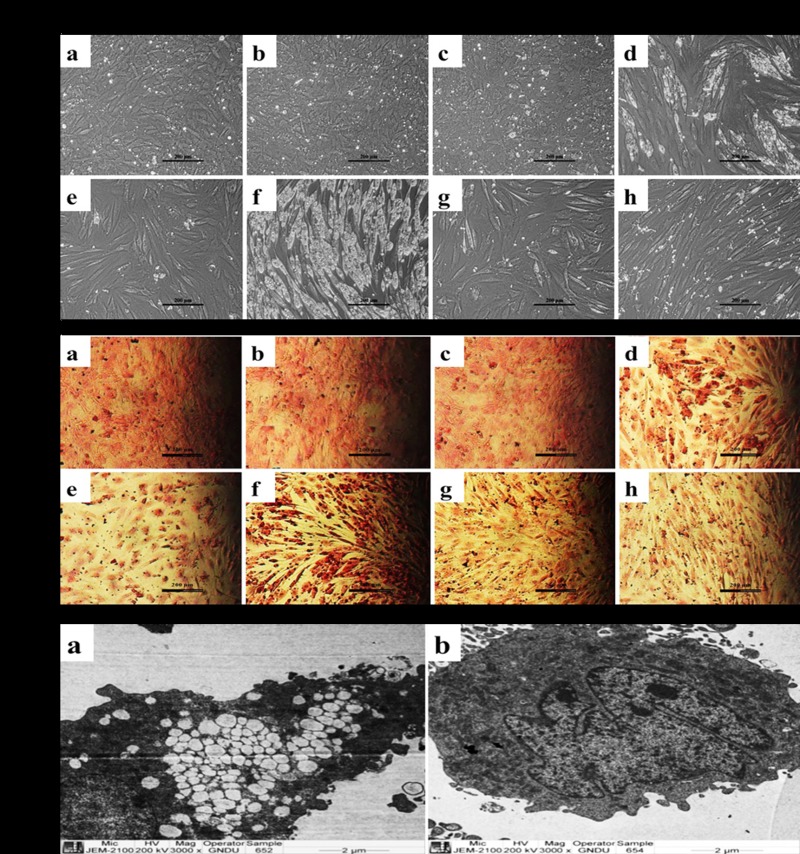
GSK3β signaling is key to adipogenic differentiation of combined treatment of CPF and RA. **(A)** Phase-contrast micrographs showing (a) CT (b) DMSO (c) LiCl (10mM) (d) RA (2μM) (e) RA+ LiCl (e) RA+ CPF (50 μM) (f) RA+ CPF+ LiCl (g) CPF **(B)** Oil Red O stained cells with similar treatment as above, indicated that LiCl completely prevented adipogenic differentiation of combined RA and CPF treatment. Scale- 200 μm. **(C)** Transmission electron micrographs reiterated the complete blockade of lipid accumulation following LiCl addition to the combined treatment of RA and CPF (b) in comparison to RA and CPF alone (a), suggesting GSK3β as the key molecule in the adipogenic differentiation. Scale- 3000x.

Treating cells with 10 mM LiCl which was well-tolerated by C_3_H_10_T½ cells (Fig 4A) brought a complete inhibition of lipid vesicle accumulation in cells which were differentiated to adipocyte-like cells following exposure to combined treatment of RA and CPF in comparison to untreated cells. It also inhibited the lipid accumulation in the cells, which were differentiated to adipogenic lineage following RA treatment alone. However, LiCl treatment did not bring any change in the undifferentiated cells and they were similar to the cells treated with DMSO or CPF alone. These observations were further reiterated by lack of Oil Red O stained cells when LiCl was added to cells either exposed to RA combined with CPF or RA alone (Fig 4B). The TEM analysis additionally reiterated the inhibitory effect of LiCl. As shown in Fig 4C, LiCl addition to RA plus CPF treated cells completely prevented any appearance of lipid-like vesicles in comparison to RA plus CPF exposed cells which were not treated with LiCl. These observations suggested a major role played by GSK3β signaling in RA and CPF-mediated differentiation of multipotent C_3_H_10_T½ cells to adipocytic lineage.

#### 4.2 CPF together with RA activates expression of adipogenic marker genes through GSK3β signaling

**Fig 5:**
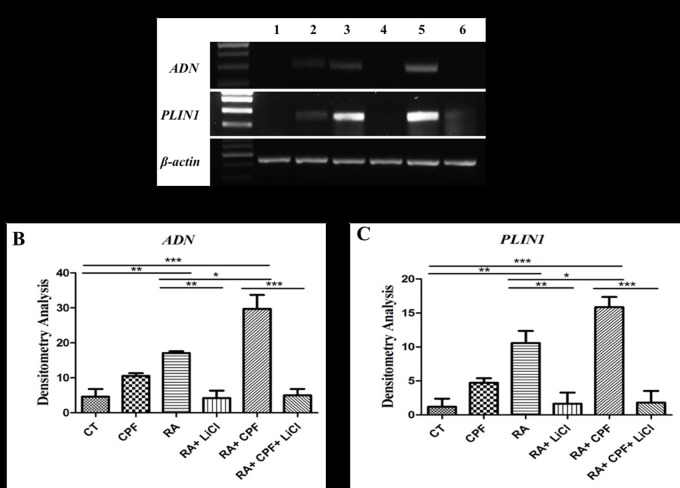
Adipogenic gene expression profile. **(a)** Electrophoretogram shows in lane (1) CT (2) 50 μM CPF (3) 2 μM RA (4) 2 μM RA+10 mM LiCl (5) 2 μM RA+50 μM CPF (6) 2 μM RA+50 μM CPF+10 mM LiCl. RT PCR results showing gene expression levels of *PLIN1* and *ADN*. *β-actin* was employed as housekeeping gene. Densitometry analysis depicting **(b)** 4, 9 and 13-fold increase in *PLIN1* gene expression of 50 μM CPF, 2 μM RA and 2 μM RA plus 50 μM CPF respectively, as compared to CT. A 2-fold increase was prevalent between RA and RA+CPF. **(c)** *ADN* gene expression was up regulated 2, 4 and 6-fold in the same samples. Between RA and RA+CPF, 2-fold upregulation was observed. Treatment with LiCl resulted in complete inhibition of gene expression of *PLIN1*as well as *ADN*. mRNA expression was normalized to *β-actin* gene expression (*p< 0.001).

Since GSK3β involvement was shown to mediate CPF and RA-induced adipogenic lineage, the effect on the adipogenic marker genes, *PLIN1* and *ADN* was studied. For this RT PCR analysis was carried out following exposure of C_3_H_10_T½ cells with 2 μM RA and/or 50 μM CPF for 20 days. The electrophoretogram in Fig 5A demonstrated that compared to CT cells (lane 1) the cells treated with 50 μM CPF (lane 2), 2 μM RA (lane 3) and 2 μM RA plus 50 μM CPF (lane 5) resulted in intensification of a band corresponding to ~247 bp corresponding to *PLIN1* and ~294 bp akin to *ADN*. The densitometry analysis reflected about 4, 9 and 13-fold increase in expression levels of the *PLIN1* gene (Fig 5B) and about 2, 4 and 6-fold increase in the *ADN* gene expression as compared to the CT (lane 1) (Fig 5C). Treatment of the cells with LiCl, exposed to RA alone (Fig 5B, lane 4) or in combination with CPF (Fig 5B, lane 6) completely abolished the expression of genes, *PLIN1* and *ADN*. These observations clearly demonstrated that CPF in combination with RA caused differentiation of C_3_H_10_T½ cells to adipogenic lineage through GSK3β signaling.

## Discussion

The cell differentiation offers a unique paradigm to understand the effects of different chemicals in target and non-target organisms [40–42]. The efficacy and consequences of many of these compounds, that mammalian system gets exposed to, have comprehensively been ascertained in the present study employing multipurpose MSCs, the C_3_H_10_T½ cells. The observations of the present study lend evidence that RA directs the undifferentiated C_3_H_10_T½ MSC-like cells to undergo adipogenic differentiation which gets accentuated in presence of CPF, an OP pesticide. This effect was discerned on day 20 when the cells attained terminal differentiation as observed by complete occupancy of lipid vesicles in the intra-cytoplasmic space. This lipidogenic effect occurred at the concentrations of CPF ranging from 0.01–50 μM, which were found to be well tolerated without causing any cytotoxicity in the undifferentiated cells. Beyond these concentrations, CPF was found to be cytotoxic within 24 hrs of exposure. Our selection of CPF doses has indeed been validated based on the biomonitoring and pharmacokinetic data reporting that concentrations upto 10 μM correspond to environmental human exposure and those higher than 100 μM reflect acute accidental exposure [43]. Therefore it is important to mention here that the concentration range of CPF that we exploited in this study actually represent real time scenario of human exposure. Moreover, being the fourth highly consumed pesticide together with its detection in bodily fluids [44], it necessitated a comprehensive study to discern the effects that it may ensue either alone or through interaction with other chemicals, like RA in the present study. Both these aspects are crucial as the CPF concentrations simulating those of environmental exposure to human beings are, whereas, the cause of concern, the lower concentrations can equally generate same concern owing to their disposition through interaction with other chemical that have been exploited in this study.

Being lipid soluble molecules, the levels of both RA and CPF remain unwantedly maintained for long time [44–46]. The outcome of this effect would mean equally likely chances of their interaction with each other and also with other lipid soluble chemicals which one may get exposed to accidently or otherwise. Such a scenario is indeed a matter of grave concern as adipose tissue is one of the important reservoirs of MSCs. And since these cells are required owing to the activation of normal wear and tear process, the accumulation of these noxious chemicals would hence interfere with MSC function and may divert the differentiation towards more adipogenic outcome. RA has previously been known to induce osteoporosis and increase the susceptibility of osteoporotic fractures by inducing bone resorption through the increased RANKL/OPG ratio mediated by RARs [32, 47, 48]. The increased adipogenic signaling potentially seems to be the factor for osteogenic inhibition [25, 49, 50]. Although only the adipogensis and not the osteogenic involvement was targeted, but it does suggest that owing to indiscriminate use of CPF together with exhaustive RA supplementation program common to many countries [47, 48, 51, 52] may produce similar outcome. Keeping this aspect in mind, the concentration of RA used in the experimental set up turned out to be in the supra-physiological range (0.1–10 μM), that mimicked the pathophysiological condition of hypervitaminosis A, which is one of the contributory factor for osteoporosis [28, 53]. At this dose level, we now show it to be an adipogenesis inducing molecule letting a judicious extrapolation for the reason to be osteoporotic. Some reports do suggest anti-adipogenic effects of RA [54], but these inhibitory effects were reported in light of the adipogenic hormonal cocktail comprising of DMI. On the contrary, our present results highlight its role when present solely in the media. Moreover, RA is also known to exert variable effects, stimulatory when introduced at an early phase of adipocyte differentiation and inhibitory at a later stage [54]. The observation of the present study, however, points towards RA stimulatory effects. The accumulation of lipid vesicles following RA exposure by 20^th^ day provided us two unique aspects of this study. One that it requires a threshold level before committing the C_3_H_10_T½ cells to adipogenic differentiation. As within two days of its exposure we did observe cells undergoing elongated morphological change. The second was that unlike conventional morphology of differentiated adipocytes that show rounded morphology filled with lipid vesicles, in the present study, we did not observe such morphology rather the lipid vesicles accumulated in elongated differentiated cells. Such adipogenic response of these undifferentiated cells to higher concentrations of RA may turn out to be a valid reason for imbalance of differentiation, thus justifying the reported role of hypervitaminosis A in the pathophysiology of osteoporosis [47, 48, 55, 56]. And this adipogenic differentiation got further augmented when CPF was also present along with RA. These effects were further reiterated by the fact that these combination treatments also led to simultaneous increase in the adipogenic marker genes like *PLIN1*, a specific protein for terminally differentiated adipocytes which is associated with the regulation of triglyceride hydrolysis in cells [57, 58] and *ADN*, a cytokine secreted by mature adipocytes [59–60]. For this adipogenic fate, it seems that threshold levels of both these genes are mandatory. This probably seems to be the case as when CPF was administered alone even though there was more intensification of bands corresponding to *PLIN1* and *ADN* in comparison to control, but their level was far less than as seen for RA or combined dose of CPF and RA exposure.

These observations besides providing the role of differentiation as toxicological knowhow paradigm also help to discern the outcome of the adverse effects following chemical interactions. This is indeed valid, as even though association of CPF with lipid metabolism is well known, yet, many contrasting effects have been observed in different model systems when this chemical has been studied in isolation [15, 61]. It is believed that these variations may be prevalent partly due to their interaction with the chemical in question and partly to the cell type used [62–64].

Unlike, as observed with RA, the CPF exposure with the conventional model of adipogenesis rather decreased the lipid accumulation in a dose dependent fashion (0.01–100 μM). These observations, thus clearly suggested that it is the interaction of CPF with the other chemicals that determines the fate rather its universal effects *per se*. Moreover, it is important to mention here that these biphasic effects of CPF (independent *vs* combined effect with RA) are indeed legitimate effects, as we did not observe any loss of cell viability with CPF concentrations ranged from 0.01–50 μM. Additionally, the observed morphological changes as mentioned above (rounded *vs* elongated) also lend credibility to such variations in the effects of CPF.

Like the known OP’s, CPF is known to exert its effect through cholinesterase inhibition [65–67]. Whether the adipogenic augmentation effect of CPF on RA-induced adipogenesis as seen in the present study also proceeds through ACh receptors certainly warrants additional study. However, the fact that C_3_H_10_T½ cells undergo adipogenic differentiation in response to minimal dose of CPF, which otherwise are well tolerated under *in vivo*, in combination with RA indeed raise serious health concerns. Such effects of CPF can be attributed to localization of drug metabolizing enzymes, cytochrome P450 on MSCs [15], which render CPF effective even at a dose as low as 0.01 μM. Metabolism of CPF to oxonic metabolites by cytochrome system of MSCs suggestively makes them vulnerable even at very low dose levels in presence of RA. Whether RA induces cytochrome P450 that increases the CPF vulnerability to be more adipogenic though have not currently been established but it certainly raises additional possibility. As a result, CPF when added alone to the C_3_H_10_T½ cells in culture remained refractory to cell differentiation and rather prevented adipogenesis of the adipogenic cocktail. Whereas, it’s combined exposure with RA resulted in a strong augmentation in the adipogenic fate even at a dose as low as 0.01 μM. Under *in vivo* state the fate of MSCs though seem to depend upon the need and the requirement but the net outcome is akin to stimulation of one lineage over the other. It is now believed that stimulation of adipogenesis appears to suppress the osteogenesis and vice versa [68]. An inverse correlation between the osteogenic and adipogenic differentiation, thus, may suggest a fine line of cell’s commitment with terminal differentiation as an outcome of local and exogenous factors, and currently, this fate was dependent upon the nature of the chemical and its interacting partners in question. This is indeed valid as the interaction of CPF with other chemical moiety viz. dexamethasone inclined the fate towards the enhanced neurodifferentiation into the dopamine phenotype [63, 64] rather to the adipogenic fate, ofcourse, realizing that cell type used in the study does impact the outcome.

Based on these observations, a reciprocal relation between adipogenic and osteogenic state is thus reiterated. This aspect though requires an additional confirmation but the fact that addition of LiCl, an inhibitor of glycogen synthase kinase 3 beta (GSK3β) [69–74], completely abolished the combined effect of CPF-RA or RA alone towards adipogenic differentiation of C_3_H_10_T½ cells conform to such a cell differentiation reciprocity. LiCl has been used as a drug for the treatment for osteoporotic patients [71]. Moreover, reduction in bone marrow adiposity and enhancements in osteocytic responses suggested that GSK3β influences MSC fate [72]. LiCl being a potent inhibitor of GSK3β, and complete blockade of lipid vesicle accumulation, Oil Red O stain and downregulation of *PLIN1* and *ADN* genes to almost non-detectable levels in the present study, following CPF and RA combined or RA alone, thus obviously linked this effect to be mediated through activation of GSK3β signaling. The activation GSK3β signaling involves an upstream inhibition of the Wnt signaling [74, 75]. The expected activation of GSK3β, due to LiCl effect, by combined CPF and RA and RA alone, thus seems to target multiple pathways involving inhibition of Wnt or regulation of frizzled receptors, involvement of G-proteins and activation of casein kinase etc. The Wnt pathway is considered a potent inhibitor of adipogenesis, preventing the *in vitro* terminal differentiation of preadipocytes [75, 76]. RA has long been shown to down regulate the Wnt signaling [75–77], thus strongly supporting that the observed effects in the present study indeed ascribe to such a pathway.

Thus, such an imbalance in the differentiating fate of cell types, as seen in the present study raises major concern as this would mean a compromise at the natural wear and tear process besides forcing the cell to commit to an unnatural cell fate. A strong association between pesticide exposure and skeletal defects in agricultural workers [16], beacon for discerning implications of OPs on cell differentiation. As the present observations suggest, this effect of OPs should not be considered an effect in isolation but rather a concern out of their interaction with other chemicals. These observations thus form an important basis for using C_3_H_10_T½ cells as a model system for the *in vitro* toxicology analysis, more so when drug-drug interactions studies are in question. Based on the observations of the present study it is concluded that crosstalk of CPF, a widely used OP pesticide and RA, vitamin A metabolite, predisposes the differentiation of C_3_H_10_T½ cell line to adipogenic fate.
