# Correction: Retinoic acid exacerbates chlorpyrifos action in ensuing adipogenic differentiation of C3H10T½ cells in a GSK3β dependent pathway

**DOI:** 10.1371/journal.pone.0178999

**Published:** 2017-05-30

**Authors:** Harkirat Singh Sandhu, A. J. S. Bhanwer, Sanjeev Puri

In Figs 1–5, the figure panel labels are missing. Please see the corrected Figs 1–5 here.

**Fig 1:**
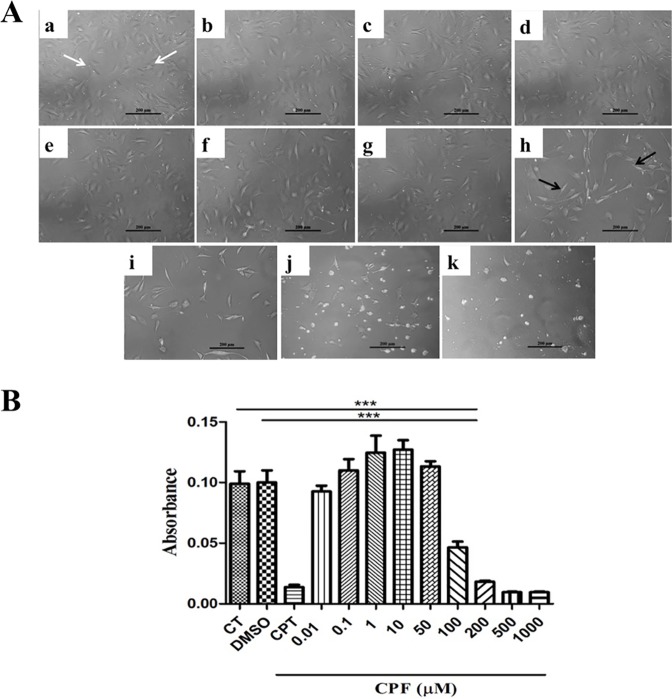
Concentration dependency of CPF on C3H10T½ MSC viability. **(A)** CPF at concentrations 0.01, 0.1, 1, 10, 50, 100, 200, 500, 1000 μM (c-k) retained the cell morphology similar to (a) control (CT, white arrowheads) and (b) vehicle (DMSO). The concentrations above 50 μM i.e. 100–1000 μM (h-k) affected the cell morphology (black arrow heads). Scale- 200 μm. **(B)** MTT assay for cell toxicity demonstrated cell viability retained till 50 μM, while a significant reduction in the cell viability was observed at concentrations beyond 50 μM (100–1000 μM). Hence, in majority of the subsequent experiments 50 μM concentration of CPF was used. Data was expressed as mean ± SEM (n = 6) and One-way ANOVA with Newman-Keuls Multiple Comparison Test performed for statistical analysis (*p<0.001).

**Fig 2:**
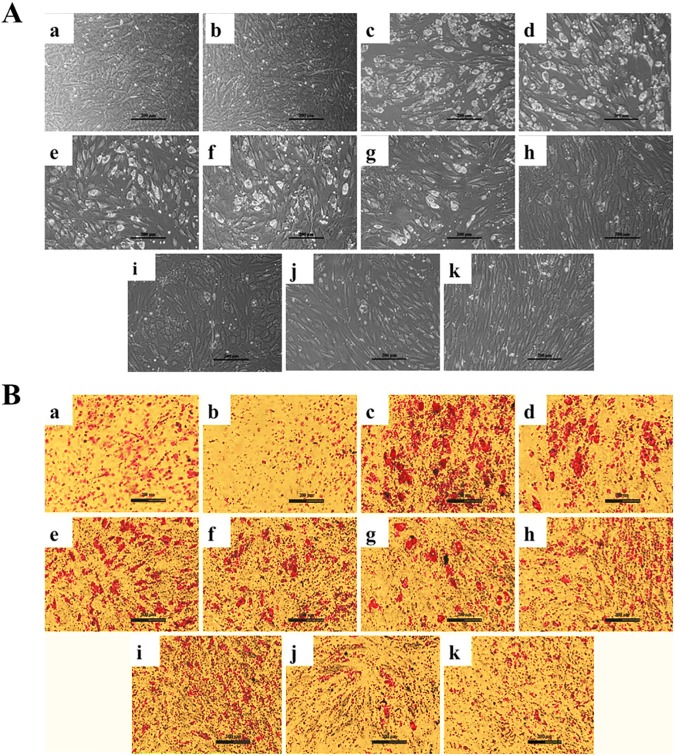
CPF alone prevented ability of adipogenic cocktail (DMI) towards differentiation of C_3_H_10_T½ cells to adipocytic lineage. **(A)** Phase-contrast micrographs indicated appearance of lipid vesicles (white) in (c) DMI and (d) DMI+ DMSO compared to (a) CT and (b) DMSO. Increasing concentrations of CPF (0.01, 0.1, 1, 10, 25, 50 and 100 μM) caused a gradual decrease in lipid vesicle accumulation of DMI-treated cells (e-k). **(B)** Oil Red O stained cells following adipogenic cocktail (DMI) treatment to C_3_H_10_T½ cells w.r.t CT and DMSO. CPF in a dose-dependent manner decreased the adipogenic differentiation (a-k, similar to above phase-contrast images). Scale- 200 μm.

**Fig 3:**
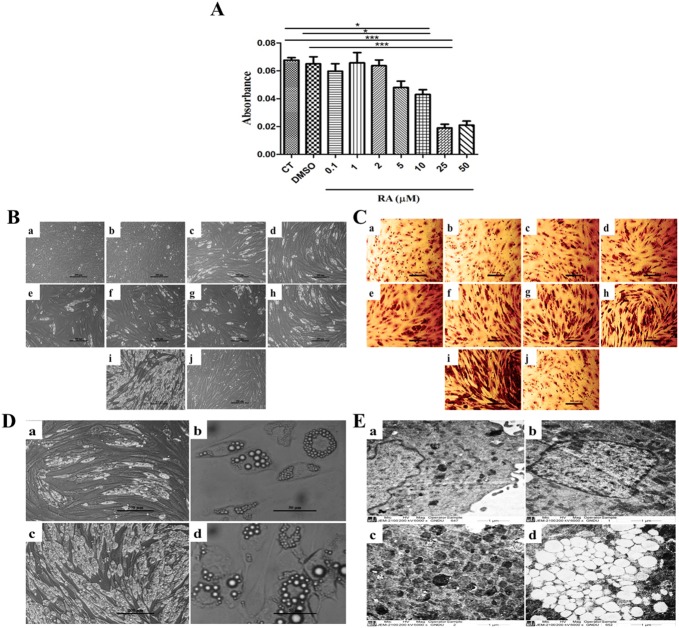
CPF augmented RA-induced differentiation of C_3_H_10_T½ cells to adipocytic lineage. **(A)** MTT assay shows cell viability retained by RA at concentrations ranging from 0.1–2 μM. Higher concentrations were found to be toxic. Hence, RA at concentration of 2 μM was used in all the subsequent experiments. Plotted values represent mean ± SEM (n = 3) and One-way ANOVA with Newman-Keuls Multiple Comparison Test performed for statistical analysis (*p<0.001). **(B)** Phase-contrast micrographs indicated appearance of lipid vesicles (white) in (c) RA in comparison to (a) CT, (b) DMSO and (j) CPF. Increasing concentrations of CPF (0.01, 0.1, 1, 10, 25, 50 μM) along with RA (2 μM) resulted in enhancement of lipid vesicles (d-i). Scale- 200 μm. **(C)** Oil Red O stained cells following above treatment. Scale- 50μm. These observations were further strengthened by **(D)** higher magnifications (a) RA (100x) (b) RA (400x) (c) RA+ CPF (100x) (d) RA+ CPF (400x). (E) 6000x transmission electron micrographs showing augmentation of lipid vesicles in combined treatment of CPF and RA (d) but considerably less in presence of RA alone (c). (a) CT and (b) CPF did not show appearance of any such lipid vesicles. Scale- 6000x.

**Fig 4:**
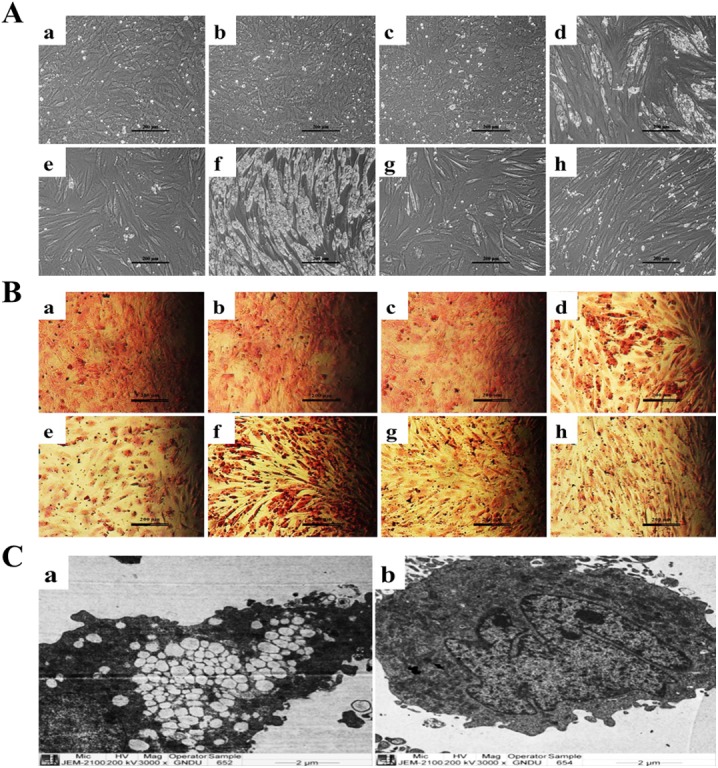
GSK3β signaling is key to adipogenic differentiation of combined treatment of CPF and RA. **(A)** Phase-contrast micrographs showing (a) CT (b) DMSO (c) LiCl (10mM) (d) RA (2μM) (e) RA+ LiCl (e) RA+ CPF (50 μM) (f) RA+ CPF+ LiCl (g) CPF **(B)** Oil Red O stained cells with similar treatment as above, indicated that LiCl completely prevented adipogenic differentiation of combined RA and CPF treatment. Scale- 200 μm. **(C)** Transmission electron micrographs reiterated the complete blockade of lipid accumulation following LiCl addition to the combined treatment of RA and CPF (b) in comparison to RA and CPF alone (a), suggesting GSK3β as the key molecule in the adipogenic differentiation. Scale- 3000x.

**Fig 5:**
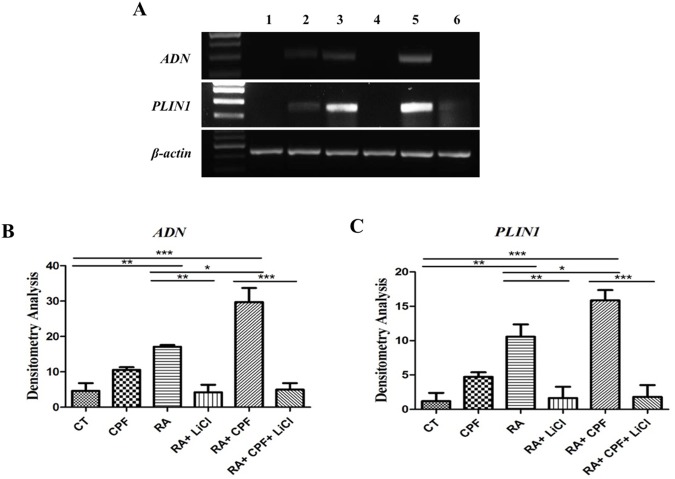
Adipogenic gene expression profile. **(a)** Electrophoretogram shows in lane (1) CT (2) 50 μM CPF (3) 2 μM RA (4) 2 μM RA+10 mM LiCl (5) 2 μM RA+50 μM CPF (6) 2 μM RA+50 μM CPF+10 mM LiCl. RT PCR results showing gene expression levels of *PLIN1* and *ADN*. *β-actin* was employed as housekeeping gene. Densitometry analysis depicting **(b)** 4, 9 and 13-fold increase in *PLIN1* gene expression of 50 μM CPF, 2 μM RA and 2 μM RA plus 50 μM CPF respectively, as compared to CT. A 2-fold increase was prevalent between RA and RA+CPF. **(c)** *ADN* gene expression was up regulated 2, 4 and 6-fold in the same samples. Between RA and RA+CPF, 2-fold upregulation was observed. Treatment with LiCl resulted in complete inhibition of gene expression of *PLIN1* as well as *ADN*. mRNA expression was normalized to *β-actin* gene expression (*p< 0.001).
